# Upregulated dual oxidase 1-induced oxidative stress and caspase-1-dependent pyroptosis reflect the etiologies of heart failure

**DOI:** 10.1186/s12860-024-00506-8

**Published:** 2024-05-15

**Authors:** Yan Song Li, Jingwen Xia, Chang Yuan Chen, Shu Hong Ren, Mao Rong He

**Affiliations:** 1 Department of Cardiovasology, Shanghai Songjiang District Center Hospital, NO.748, Zhongshan Middle Road, Songjiang District, Shanghai, 201600 China; 2 Department of Cardiovasology, Shanghai Baoshan District Luodian Hospital, No. 88 Yongshun Road, Baoshan District, Shanghai, 201908 China

**Keywords:** Reactive oxygen species, Dual oxidase 1, Heart failure, Pyroptosis

## Abstract

**Background:**

Oxidative stress is implicated in the pathogenesis of heart failure. Dual oxidase 1 (DUOX1) might be important in heart failure development through its mediating role in oxidative stress. This study was designed to evaluate the potential role of DUOX1 in heart failure.

**Materials and methods:**

AC16 cells were treated with 2 µmol/L of doxorubicin (DOX) for 12, 24, and 48 h to construct a heart failure model. DUOX1 overexpression and silencing in AC16 cell were established. DUOX1 expression was detected by Quantitative real-time polymerase chain reaction (qRT-PCR) and western blot. Pyroptosis and reactive oxygen species (ROS) production were measured by flow cytometry.

**Results:**

Increased DUOX1 expression levels were observed after DOX treatment for 24 h in AC16 cells. DUOX1 silencing inhibited DOX-induced pyroptosis and ROS production. The release of IL-1β, IL-18, and lactate dehydrogenase (LDH), and expression levels of pyroptosis-related proteins were also decreased. DUOX1 overexpression increased pyroptosis, ROS production, IL-1β, IL-18, and LDH release, and pyroptosis-related protein expression. N-acetyl-cysteine (NAC) significantly reversed DUOX1-induced pyroptosis, ROS, and related factors.

**Conclusion:**

These results suggest that DUOX1-derived genotoxicity could promote heart failure development. In the process, oxidative stress and pyroptosis may be involved in the regulation of DUOX1 in heart failure.

**Supplementary Information:**

The online version contains supplementary material available at 10.1186/s12860-024-00506-8.

## Introduction

Heart failure represents the last stage of various diseases caused by myocardial myofibrillar contractile dysfunction with a syndrome leading to cardiac impairment, and the disease has been described as an emerging epidemic for 25 years [1]. Although the survival and pharmacological treatment of the disease have been improved, the prevalence of heart failure remains rising as a result of a growing and aging population [2, 3]. If a timely treatment is not available, it will pose a serious threat to the patient’s life and safety. An effective and timely heart failure treatment deserves great attention from clinical medical workers.

It is reported that oxidative stress was one of the main causes of heart failure development [4]. Oxidative stress is described as a condition of excess reactive oxygen species (ROS) relative to antioxidant defense and ROS is known to play a prominent role in the pathogenesis of various cardiac disorders, such as myocardial infarction and heart failure [5]. Study has revealed that ROS production is associated with macrophage inflammasome activation, which may directly cause cellular damage, oxidative stress, and pyroptosis, and uncontrolled ROS production-induced pyroptosis has been observed in various diseases [6–8]. Pyroptosis is a highly regulated cell death process accompanied by the release of cellular contents and pro-inflammatory mediators, and is mainly mediated by various caspases, including caspase-1 [9–11]. Caspase-1-dependent pyroptosis, a NOD-like receptor family pyrin domain containing 3 (NLRP3)-mediated classical pathway of pyroptosis, could lead to the activation of NLRP3 inflammasome and downstream caspase-1, IL-1β, and IL-18 [11]. Mature IL-1β is a pro-inflammatory mediator that mainly modulates the adaptive immune cells by recruiting innate immune cells to infection sites. Mature IL-18 is significantly related to interferon-γ production [12]. The functional role of pyroptosis in cardiovascular diseases (CVDs) has been widely reported [7, 13, 14].

NAPDH oxidases (NOXs) belong to the family of flavoenzymes, which included a number of isoforms, NOX1-5, and dual oxidases 1 and 2 (DUOX1-2). NOXs play a critical role in redox signaling and various CVDs [15]. NADPH oxidases are traditionally known as one of most important proteins associated with ROS generation in tissues and cells [16]. For example, in the heart, NOX 2 and 4 are the major sources of O_2_^-^and H_2_O_2_, thereby playing roles in ROS production and consequent cardiac injury [17, 18]. NOXs are superoxide (O_2_^－^)-generating enzymes that catalyze the electron transfer from NADPH to molecular O_2_ [16], and the potential role of NOX1 and NOX2 in controlling reperfusion damage were observed [19]. DUOX1 is important in cell signaling, hypoxic response, and immune function [20]. For example, the upregulation of *Duox1* and *Duox2* can induce chronic oxidative stress and fibrosis [21]. Additionally, *Duox1* knockdown promotes wound healing by decrease ROS production [22]. However, the role of DUOX1 in heart failure is complex and remains unclear.

Various studies focused on the effect of ROS on heart failure, but only few studied the role of DUOX1. Therefore, in order to evaluate the potential role of DUOX1 in heart failure, an in vitro model of heart failure using doxorubicin (DOX) was constructed. DOX is one of the most effective and broad-spectrum anthracycline antibiotics [23], and it is mainly suitable for acute leukemia, and is effective for both acute lymphoblastic leukemia and granulocytic leukemia. It is generally used as a second-line drug, that is, this drug can be considered when the first-line drug is resistant [24, 25]. As for malignant lymphoma, it can be used as the drug of choice for alternate use. It also has a certain effect on breast cancer, sarcoma, lung cancer, bladder cancer and other cancers, and they are often used in combination with other anticancer drugs [26]. Additionally, it can induce excessive ROS production in cardiac mitochondria, leading to acute cardiotoxicity and irreversible degenerative cardiomyopathy [23]. DOX is reported to be widely used in inducing heart failure in animal studies [27]. DOX-induced cardiotoxicity is characterized by left ventricular dysfunction and cardiac hypertrophy, leading to congestive heart failure. Here, we attempted to demonstrate the role and mechanism of DUOX1 in regulating DOX-induced heart failure, which may provide an evidence to identify DUOX1 as a potential therapeutic target for heart failure.

## Materials and methods

### Cell culture

Human cardiomyocyte cell line AC16 was cultured in a cell incubator with 5% CO_2_ at 37 °C. Dulbecco’s Modified Eagle’s medium (DMEM, Hyclone, SH30243.01; Logan, UT, US) was supplemented with 10% fetal bovine serum (GIBCO, 16000e044; Carlsbad, CA, USA) to culture AC16 cells. Additionally, to avoid bacterial contamination, 1% penicillin with streptomycin (Solarbio, Beijing, China) was added in the DMEM medium. For heart failure model, AC16 cells were treated with DOX (Sigma-Aldrich, 25316-40-9) at 2 µmol/L for 12, 24, and 48 h. The mRNA level of DUOX1 was quantified using quantitative real-time polymerase chain reaction (qRT-PCR), and the protein levels of DUOX1 were assessed by western blot assay.

### Construction of vectors with DUOX1 overexpression

The primers for DUOX1 (NM_017434.5) containing EcoR I and BamH I were designed, and DUOX1 was synthesized based on the designed primer with two kinds of restriction enzyme. Then, to construct the DUOX1 overexpression plasmids, the DUOX1 after amplification was implanted into the vector of pLVX-Puro (Clontech).

After the cells grew to 90% confluency, transfection was conducted. Based on the manufacturer’s instruction, pLVX-Puro-DUOX1, psPAX2, and pMD2G (Addgen) were co-transfected into 293T cells using Lipofectamine™ 2000 (Invitrogen, CA, USA).

The primers for DUOX1 were designed as follows:

DUOX1-F: 5′-CGGAATTCATGGGCTTCTGCCTGGC-3′ (EcoR I).

DUOX1-R: 5′-CGGGATCCCTAGAAGTTCTCATAATGGTGGGAG-3′ (BamH I).

### Construction and transfection of DUOX1 silencing vectors

A siRNA sequence targeting DUOX1 was synthesized and inserted into the PLKO.1 vector. The lentiviral production system included the expression vectors psPAX2, pMD2G, and pLKO.1-shDUOX1, which were all co-transfected into 293T cells (ATCC) and packaged. Meanwhile, the plasmid of the scrambled shRNA was constructed as the negative control. RNA interference is a powerful tool to induce loss-of-function phenotypes by inhibiting gene expression at the post-transcriptional level. One is that dsRNA is cut into siRNA, which allows a specific mRNA to be degraded; the other is that as long as there is a long dsRNA, it can degrade all RNA and inhibit the synthesis of all proteins. The pLKO.1-puro plasmid inserted the following sequences of DUOX1 interference: DUOX1 site 1 (1842–1860): CCATCGTCCTTGAACAATT; DUOX1 site 2 (3085–3103): GGAACTGACATGGGAAGAT; and DUOX1 site 3 (4572–4590): GCTGCCAAGTGTTCTGTAA.

### qRT-PCR

To quantify the mRNA levels of DUOX1, the total RNA in cells was extracted using a TRIzol reagent (Invitrogen, 1596-026). After quantifying and confirming the RNA integrity, the RNA samples were reverse-transcribed to cDNA; then, cDNA was amplified using SYBR Green qPCR Master Mixes (Thermo Fisher, Rockford, IL, USA). The Ct value was defined as the number of cycles that the fluorescent signal in the reaction tube reaches the set threshold. The relative DUOX1 mRNA levels normalized to GAPDH were calculated using the 2^−△△Ct^ method. The primer sequences for DUOX1 were designed as follows: Primer F: 5′ AGTGCCTGGATTGTTGCC 3′ and Primer R: 5′ TTGCTGGACAGGACGAAG 3′. The primer sequences for GAPDH were designed as follows: Primer F: 5′ GGATTGTCTGGCAGTAGCC 3′ and Primer R: 5′ ATTGTGAAAGGCAGGGAG 3′.

### Western blot analysis

The total protein from AC16 cells was extracted using a RIPA buffer (Solarbio, R0010, Beijing, China), and the protein level was determined by BCA protein assay kit (Thermo Fisher Scientific, PICPI23223). The sample was boiled at 95 °C for 10 min and separated through the gels with 10% concentration of SDS-PAGE gel (JRDUN Biotechnology Co., Ltd, Shanghai, China). Then, the separated proteins were transferred to polyvinylidene fluoride membrane and blocked with 5% nonfat milk. After blocking, the membrane was further incubated with anti-DUOX1, anti-active caspase-1, anti-GAPDH (67226-1-lg, 22915-1-AP, 60004-1-1G, Proteintech), anti-apoptosis-associated speck-like protein containing a CARD (ASC), anti-NLRP3, anti-pro-caspase-1, and anti-Gasdermin D-N domain (GSDMD-N) (Ab155970, Ab263899, Ab179515, Ab215203, Abcam) overnight at 4 °C with gentle shaking. After washing thrice by TBST(TBS + Tween20), the polyvinylidene fluoride membrane was incubated with horseradish peroxidase-conjugated secondary antibodies (1:1000, Beyotime, Shanghai, China) for 1 h at 25 °C. Finally, the expression levels of proteins were measured using a chemiluminescent imaging system (Tanon 5200, Shanghai, China).

### ROS detection

To detect the ROS production, Active Oxygen Detection Kit (S0033, Beyotime) was employed. First, the cell pellets were obtained after centrifugation and resuspended using 1 mL of cooled PBS. Following the manufacturer’s instruction, the DCFH-DA staining working solution was diluted to 10 µM using a serum-free medium. Then, the DCFH-DA probe solution was added to the cell suspension in the dark for 20 min at 37 °C. To make the probe in full contact with the cells, the samples were mixed every 3–5 min for 20 min. Then, the cells were washed thrice using a serum-free medium to remove the DCFH-DA probe that did not enter the cells. Finally, the samples were detected by flow cytometry (BD Biosciences, Franklin Lake, NJ, USA).

### Pyroptosis assay

To detect the level of pyroptosis, the cells were resuspended in PBS. Then, active caspase-1 (1:30; EL900443, EterLife) was added in the cell incubation for 1 h at 25 °C. To remove non-combined caspase-1, the cells were washed with PBS for three times and incubated with propidium iodide solution (PI, P3566; Invitrogen) at 3 µM for 15 min in the dark. Finally, the rates of cell pyroptosis were estimated based on flow cytometry.

### Enzyme-linked immunosorbent assay (ELISA)

The secreted IL-1β and IL-18 in the supernatants were detected using ELISA. The cells were collected by centrifuging at 3000 rpm/min for 20 min. After removing the cell pellets, the secreted IL-1β and IL-18 were quantified using Human IL-1β and IL-18 ELISA kits (NeoBioscience, Shenzhen, Guangdong, China), following the manufacturer’s instructions. The amount of IL-1β and IL-18 was detected at 450 nm by a microplate reader (Bio-Rad, Hercules, CA, USA).

### Biochemical detection

The lactate dehydrogenase (LDH) in supernatant and cellular protein level was detected using the LDH (A020-1) Kit and BCA (A045-3) Kit (Nanjing, Jiancheng Biotechnology Research Institute, Jiangsu, China). The supernatant and kit solution were mixed for 15 min at 37 °C in a water bath. Then, the LDH and cellular protein were measured at 440 nm (LDH) and 562 nm (BCA).

### Statistical analysis

Each experiment was performed in triplicate. The data were presented as mean value ± standard deviation of three independent biological replicates. To compare the difference in mean values among the groups, one-way analysis of variance with Tukey’s post hoc tests was applied. *P* < 0.05 was regarded as statistically significant. All statistical analyses in the study were performed using the GraphPad Prism 7.0 software (San Diego, CA, USA).

## Results

### DUOX1 was highly expressed in DOX-induced AC16 cells in a time-dependent manner

**Fig. 1:**
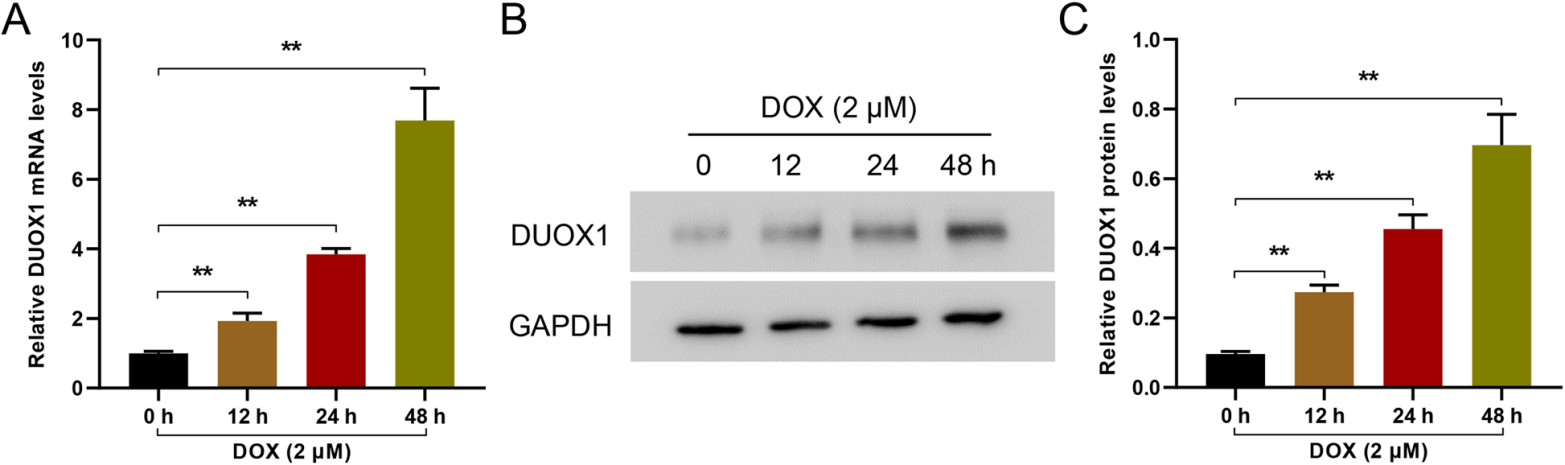
DUOX1 was dependently induced by DOX in AC16 cells. Human cardiac AC16 cells were treated with 2 µmol/L of DOX to simulate and construct an in vitro model of heart failure. qRT-PCR (**A**) and western blot (**B**) detected the expression of DUOX1 at different time points. **p < 0.01. The blots were cut prior to hybridisation with antibodies during immunoblotting experiments

Human cardiac AC16 cells were treated with 2 µmol/L of DOX to simulate and construct a heart failure model in vitro. qRT-PCR and western blot detection revealed that the expression of DUOX1 increased in a time-dependent manner (Fig. 1), that is, DUOX1 expression increased significantly as time increased. Further, 24 h was chosen as the DOX treatment time.

### DUOX1 silencing potently reversed DOX-induced pyroptosis and ROS production

**Fig. 2:**
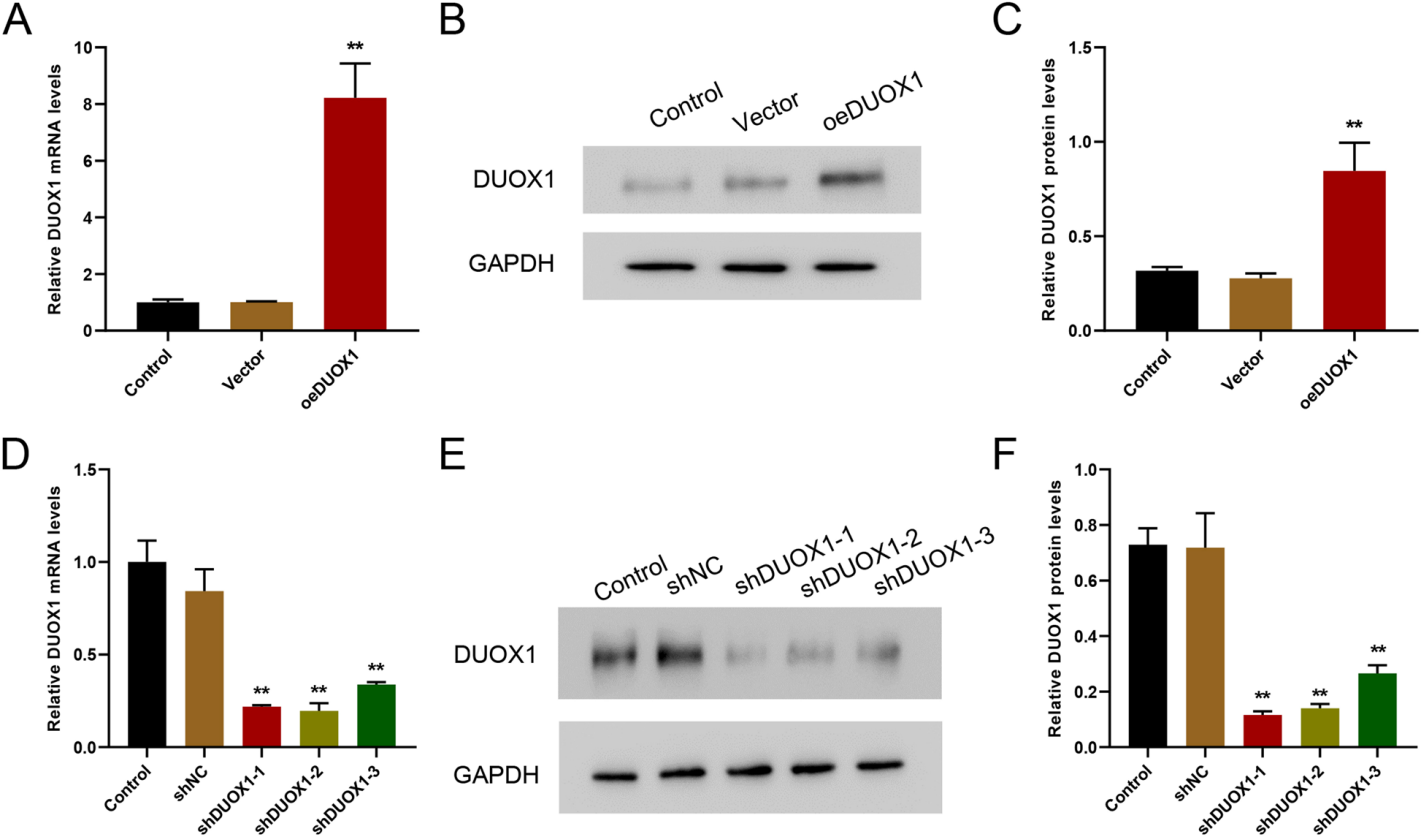
The efficiency identification of DUOX1 overexpression and knockdown. DUOX1 overexpression and interference lentiviral vector was transfected into myocardial AC16 cells, and the overexpression (**A**-**C**) and interference (**D**-**F**) efficiency of DUOX1 was detected by qRT-PCR and western blot. **p < 0.01 vs. vector/shNC. The blots were cut prior to hybridisation with antibodies during immunoblotting experiments

The overexpression and interference efficiency of DUOX1 was examined by qRT-PCR and western blot on mRNA and protein levels, respectively. Both mRNA and protein levels of DUOX1 were significantly increased in the DUOX1 overexpression (oeDUOX1) group (Fig. 2A-C), and decreased in the group of shDUOX1-1, shDUOX1-2, shDUOX1-3 as compared with shNC (Fig. 2D-F), suggesting the model of DUOX1 overexpression and silencing were successfully constructed (Fig. 2).

**Fig. 3:**
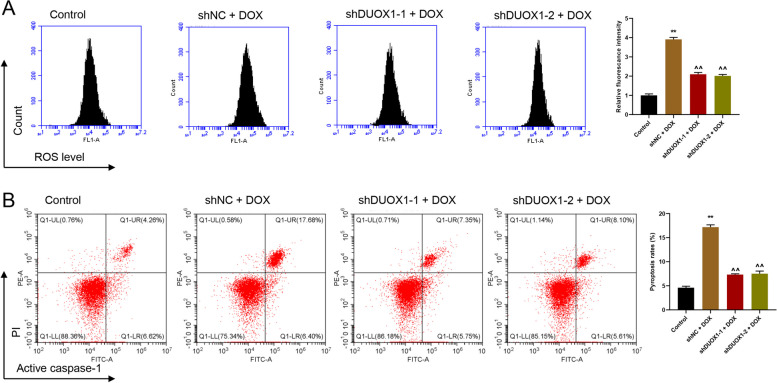
DUOX1 knockdown reversed DOX-induced ROS production and pyroptosis. **A** DUOX1 knockdown reversed DOX-induced ROS production, tested by flow cytometry. **B** DUOX1 knockdown reversed DOX-induced pyroptosis, tested by flow cytometry. **p < 0.01 vs. control; ^^*p* < 0.01 vs. shNC + DOX (2 µmol/L)

After efficiency appraisal, the shDUOX1-1 and shDUOX1-2 vector was transfected to AC16 cells, respectively. Then DOX (2 µmol/L) was added into the cells. As shown in Fig. 3, DOX treatment led to an increase ROS production (Fig. 3A) and pyroptosis (Fig. 3B) in AC16 cells. While, silencing DUOX1 significantly reversed the increased ROS production and pyroptosis introduced by DOX (Fig. 3).

### DUOX1 silencing potently reversed DOX-induced inflammation and abnormal protein expression

**Fig. 4:**
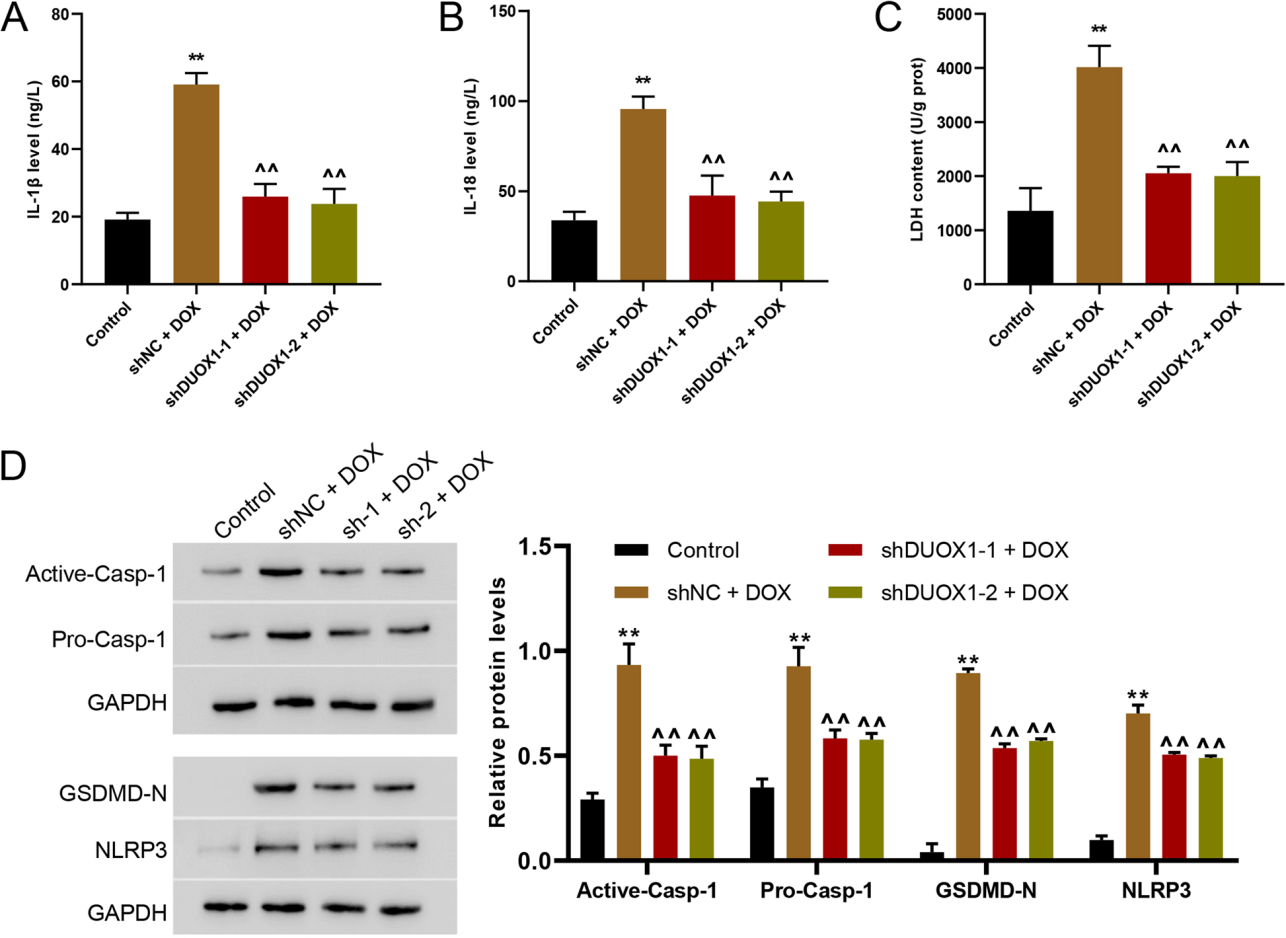
DUOX1 knockdown reversed DOX-induced inflammation and abnormal protein expression. The secretion of IL-1β (**A**) and IL- 18 (**B**) in the cell supernatant was detected by ELISA. **C** LDH activity was detected by biochemical method. **D** Western blot was used to detect the expression of pyroptosis indexes active caspase-1, pro-caspase-1, NLRP3, and GSDMD-N. **p < 0.01 vs. control; ^^*p* < 0.01 vs. shNC + DOX (2 µmol/L). The blots were cut prior to hybridisation with antibodies during immunoblotting experiments

Further, we have investigated the effects of DUOX1 knockdown on IL-1β and IL-18 secretion, and LDH release, as well as protein level of pyroptosis associated pro- and active caspase-1, GSDMD-N, and NLRP3. The results indicated that DOX treatment led to IL-1β (Fig. 4A) and IL-18 secretion (Fig. 4B). The CIK cell killing activity was detected using LDH release test, and LDH release was significantly increased after DOX treatment (Fig. 4C), suggesting the increased CIK cell activity. After DUOX1 silencing, IL-1β and IL-18 secretion, and LDH release were significantly decreased (Fig. 4A-C). In addition, the protein expression of pro- and active caspase-1, GSDMD-N, and NLRP3 was detected using western blot. Figure 4D showed that the expression levels of pro- and active caspase-1, GSDMD-N, and NLRP3 in AC16 cells were all increased after DOX treatment, and DUOX1 silencing reversed the increased levels of the mentioned proteins (Fig. 4D).

### DUOX1 promoted the pyroptosis in AC cells probably through ROS regulation

**Fig. 5:**
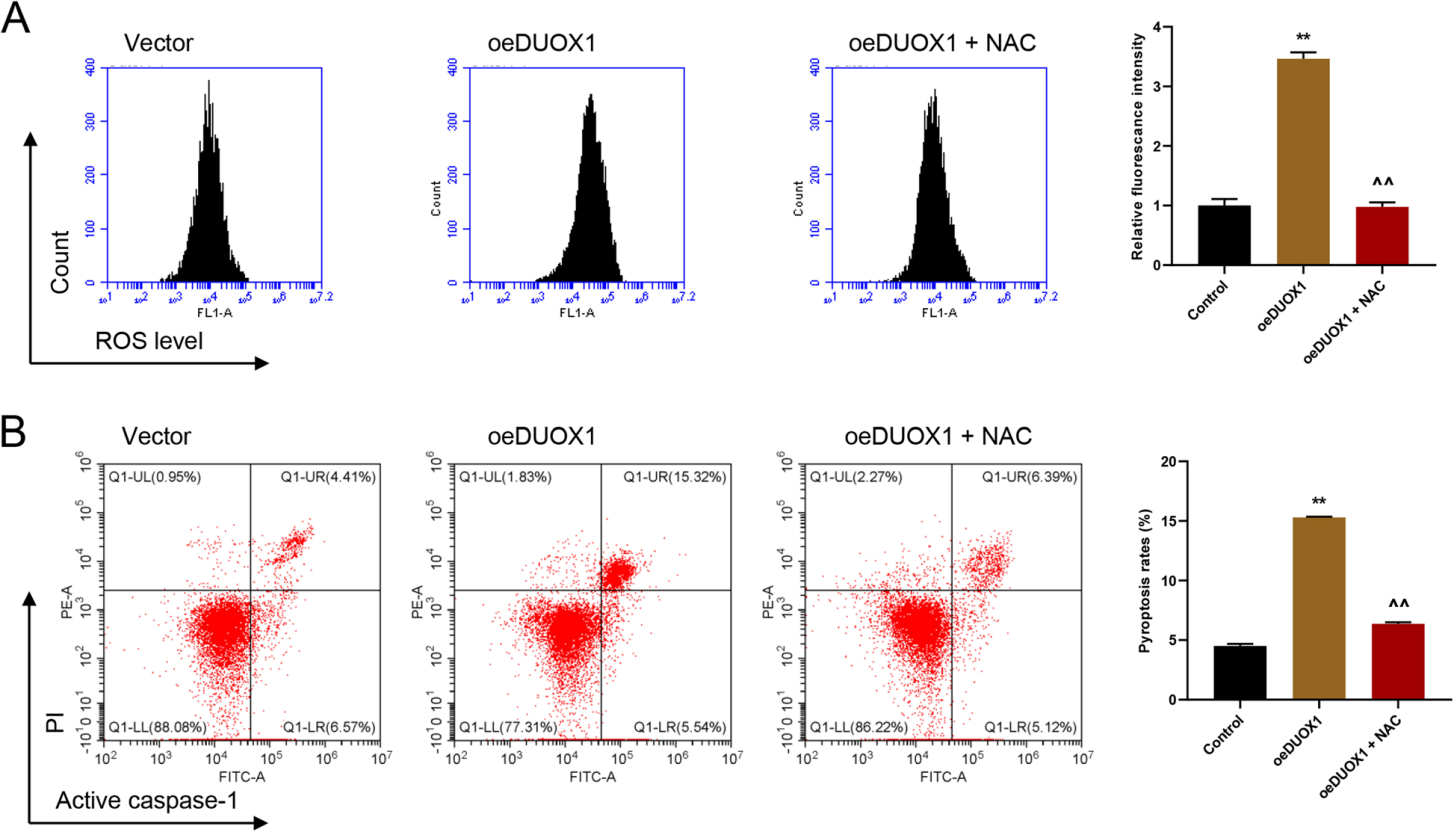
DUOX1 promoted ROS production and pyroptosis were inhibited by NAC. ROS scavenger NAC treatment reversed DUOX1 overexpression induced ROS production (**A**) and pyroptosis (**B**). Flow cytometry was designed to detect cell ROS level and pyroptosis. **p < 0.01 vs. vector; ^^*p* < 0.01 vs. oeDUOX1 + NAC (100 µmol/L)

**Fig. 6:**
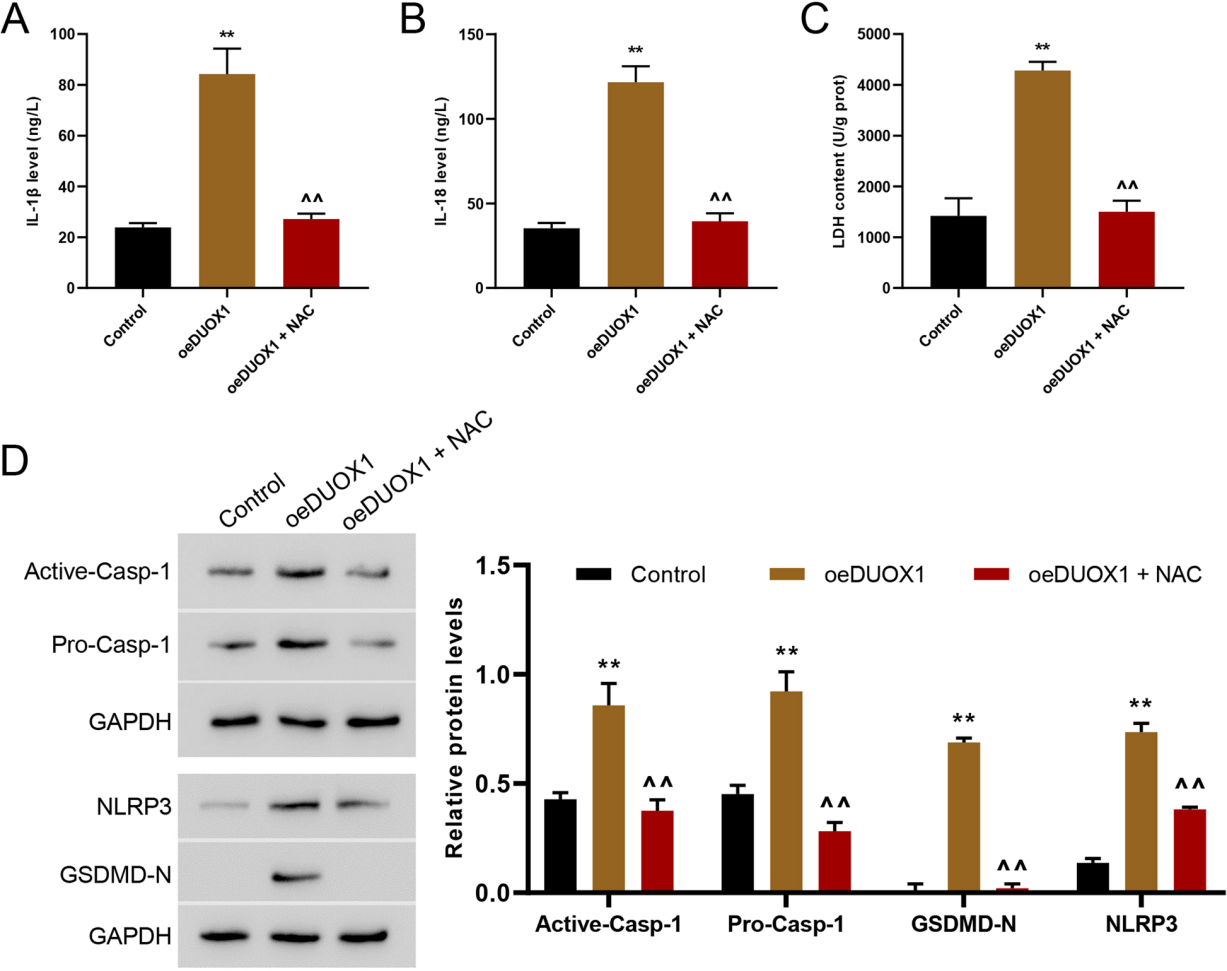
DUOX1 promoted the pyroptosis of AC16 cells probably through ROS regulation. The secretion of IL-1β (**A**) and IL-18 (**B**) in the cell supernatant was detected by ELISA. **C** LDH activity was detected by biochemical method. **D** Western blot was used to detect the expression of active caspase-1, pro-caspase-1, NLRP3, and GSDMD-N. **p < 0.01 vs. vector; ^^*p* < 0.01 vs. oeDUOX1 + NAC (100 µmol/L). The blots were cut prior to hybridisation with antibodies during immunoblotting experiments

In subsequent experiments, we have examined the effects of DUOX1 overexpression on AC16 cells, and the participation of ROS was investigated during this process. AC16 cells was treated by DUOX1 overexpression and the ROS scavenger NAC (100 µM) for 24 h, and then the ROS production, pyroptosis, IL-1β and IL-18 secretion, LDH release, and the protein expression of pro- and active caspase-1, GSDMD-N, and NLRP3 were tested. The results showed the ROS production, and pyroptosis were induced by DUOX1 overexpression (Fig. 5A, B). Meanwhile, DUOX1 overexpression increased IL-1β and IL-18 secretion, LDH release, protein levels of pro- and active caspase-1, GSDMD-N, and NLRP3 (Fig. 6A-D). Above all, these changes induced by DUOX1 overexpression were all reversed by NAC treatment (Figs. 5 and 6). Suggested that ROS mediated DUOX1 induced cell pyroptosis.

## Discussion

Up to now, heart failure remains a major cause of mortality, morbidity, and poor quality of life. To maintain the normal function of cardiovascular system, a balance between cell formation and death is needed [28]. DUOX, as a member of NADPH oxidase family, participated directly in generating hydrogen peroxide/ROS [29]. In normal condition, there is a balance between ROS and antioxidants, but the excessive amounts of ROS would result in various CVDs, including heart failure [4]. In this study, we found the increased DUOX1 expression levels in DOX-induced cellular heart failure model, suggesting that DUOX1 may contribute to heart failure development.

The underlying mechanism of DUOX1 in regulating heart failure has been also explored. It is well-known that heart failure development is associated with oxidative stress, which might be caused by the complex effects of ROS [4]. DUOX1-induced ROS production has also been shown in various diseases, such as DUOX1 nasal polyposis, allergy and asthma, chronic obstructive pulmonary disease, lung fibrosis and cancer [30]. Consistent with these reports, we found that the increased ROS production were associated with DUOX1 expression. Silencing DUOX1 potently reversed DOX-induced ROS production. Meanwhile, NAC, a kind of active oxygen inhibitor, significantly reversed the level of ROS, and related factors induced by DUOX1 overexpression, suggesting a direct oxidative stress effect of increased DUOX1 expression on heart failure.

Furthermore, the relationship between pyroptosis and pathogenesis of various CVDs, including heart failure, has been demonstrated [31], and the inhibition of pyroptosis was recognized as a potential therapeutic intervention to prevent CVDs [32]. Consistent with these, our data showed that the increased pyroptosis was associated with DUOX1 expression. Silencing DUOX1 potently reversed DOX-induced pyroptosis. From these, we speculated that pyroptosis may be involved in DUOX1 regulation in DOX-induced heart failure. The Studies also reported that elevated mRNA of NLRP3 and enhanced cleavage of caspase-1 were observed along with cardiac hypertrophy and ventricular dilatation in calcineurin transgene (CNTg) mice, an established mouse model for chronic heart failure [33]. Genetic ablation of *NLRP3* or administration of IL-1 receptor antagonist can reduce cardiac inflammation and systolic dysfunction [34–36]. In line with these previous reports, the data in this study showed the increased caspase-1 expression when the level of pyroptosis was high. Caspase-1 is one of the pro-inflammatory family of caspases, is a necessary ingredient in the activation of pro-IL-1β and pro-IL-18 [37]. In the pathophysiology of heart failure, previous data demonstrated the critical roles of active IL-1β and IL-18 [38, 39]. This study showed the increased secretion of IL-1β and IL-18 when DUOX1 was overexpressed in AC16 cells, suggesting that the caspase-1-dependent pyroptosis in heart failure was triggered by the upregulation of DUOX1. Meanwhile, NAC significantly reversed the levels of pyroptosis, and related factors induced by DUOX1 overexpression, suggesting that DUOX1 regulates caspase-1-dependent pyroptosis in AC16 cells may through modulating ROS.

NLRP3-mediated pyroptosis pathway are widely studied, and related inhibitors are well used in clinical practice in cardiovascular disease. Our results illustrate that suppressing ROS production by DUOX1 knockdown was an effective strategy to decrease NLRP3-mediated myocardial cells injury. These suggest that similar function of DUOX1 are very likely reproduced in other cardiovascular disease, such as myocardial ischemia-reperfusion injury and myocardial infarction. Meanwhile, the role of DUOX1 in myocardial injury and ventricular remodeling needs more practice and exploration.

## Conclusions

Our study revealed the important role of DUOX1 in heart failure. DUOX1 inhibition in heart failure might prevent the damage induced by oxidative stress and pyroptosis, which might be a novel therapeutic biomarker for heart failure. However, our conclusion was based on the experiment in vitro. Future studies on animal model and primary myocardial cells should be designed to provide more insight into the biological pathways associated with DUOX1 underlying heart failure.

### Supplementary information

**Supplementary Material 1.**

## Data Availability

The data are available from the corresponding author upon reasonable request.

## References

[1] Savarese G, Becher PM, Lund LH, Seferovic P, Rosano GMC, Coats AJS (2023). Global burden of heart failure: a comprehensive and updated review of epidemiology. Cardiovasc Res.

[2] Odegaard KM, Hallen J, Lirhus SS, Melberg HO, Halvorsen S (2020). Incidence, prevalence, and mortality of heart failure: a nationwide registry study from 2013 to 2016. ESC Heart Fail.

[3] Ren QW, Li XL, Fang J, Chen Y, Wu MZ, Yu YJ, Liao SG, Tse HF, Yiu KH (2020). The prevalence, predictors, and prognosis of tricuspid regurgitation in stage B and C heart failure with preserved ejection fraction. ESC Heart Failure.

[4] Ng ML, Ang X, Yap KY, Ng JJ, Goh ECH, Khoo BBJ, Richards AM, Drum CL (2023). Novel oxidative stress biomarkers with risk prognosis values in heart failure. Biomedicines.

[5] Qiu Z, He Y, Ming H, Lei S, Leng Y, Xia ZY (2019). Lipopolysaccharide (LPS) aggravates high glucose- and hypoxia/reoxygenation-induced injury through activating ROS-dependent NLRP3 inflammasome-mediated pyroptosis in H9C2 Cardiomyocytes. J Diabetes Res.

[6] Zheng D, Liu J, Piao H, Zhu Z, Wei R, Liu K (2022). ROS-triggered endothelial cell death mechanisms: focus on pyroptosis, parthanatos, and ferroptosis. Front Immunol.

[7] Tian K, Yang Y, Zhou K, Deng N, Tian Z, Wu Z, Liu X, Zhang F, Jiang Z (2023). The role of ROS-induced pyroptosis in CVD. Front Cardiovasc Med.

[8] Zhou X, An B, Lin Y, Ni Y, Zhao X, Liang X (2023). Molecular mechanisms of ROS-modulated cancer chemoresistance and therapeutic strategies. Biomed Pharmacother.

[9] Vasudevan SO, Behl B, Rathinam VA (2023). Pyroptosis-induced inflammation and tissue damage. Semin Immunol.

[10] Zhou J, Qiu J, Song Y, Liang T, Liu S, Ren C, Song X, Cui L, Sun Y (2023). Pyroptosis and degenerative diseases of the elderly. Cell Death Dis.

[11] Fu J, Wu H (2023). Structural mechanisms of NLRP3 inflammasome assembly and activation. Annu Rev Immunol.

[12] Kupz A, Pai S, Giacomin PR, Whan JA, Walker RA, Hammoudi PM, Smith NC, Miller CM (2020). Treatment of mice with S4B6 IL-2 complex prevents lethal toxoplasmosis via IL-12- and IL-18-dependent interferon-gamma production by non-CD4 immune cells. Sci Rep.

[13] Yarovinsky TO, Su M, Chen C, Xiang Y, Tang WH, Hwa J (2023). Pyroptosis in cardiovascular diseases: pumping gasdermin on the fire. Semin Immunol.

[14] Hu B, Chen W, Zhong Y, Tuo Q (2023). The role of lncRNA-mediated pyroptosis in cardiovascular diseases. Front Cardiovasc Med.

[15] Augsburger F, Filippova A, Rasti D, Seredenina T, Lam M, Maghzal G, Mahiout Z, Jansen-Durr P, Knaus UG, Doroshow J (2019). Pharmacological characterization of the seven human NOX isoforms and their inhibitors. Redox Biol.

[16] Ogboo BC, Grabovyy UV, Maini A, Scouten S, van der Vliet A, Mattevi A, Heppner DE (2022). Architecture of the NADPH oxidase family of enzymes. Redox Biol.

[17] Pena E, Brito J, El Alam S, Siques P (2020). Oxidative stress, kinase activity and inflammatory implications in right ventricular hypertrophy and heart failure under hypobaric hypoxia. Int J Mol Sci.

[18] Rajaram RD, Dissard R, Jaquet V, de Seigneux S (2019). Potential benefits and harms of NADPH oxidase type 4 in the kidneys and cardiovascular system. Nephrol Dial Transpl.

[19] Bkaily G, Najibeddine W, Jacques D (2019). Increase of NADPH oxidase 3 in heart failure of hereditary cardiomyopathy (1). Can J Physiol Pharmacol.

[20] Kolivand S, Amini P, Saffar H, Rezapoor S, Najafi M, Motevaseli E, Nouruzi F, Shabeeb D, Eleojo Musa A (2019). Selenium-L-methionine modulates radiation injury and Duox1 and Duox2 upregulation in rat’s heart tissues. J Cardiovasc Thorac Res.

[21] Farhood B, Aliasgharzadeh A, Amini P, Saffar H, Motevaseli E, Rezapoor S, Nouruzi F, Shabeeb D, Musa AE, Ashabi G (2019). Radiation-induced dual oxidase upregulation in rat heart tissues: protective effect of melatonin. Med (Kaunas Lithuania).

[22] Wang H, Lu H, Wu Y (2021). Knockdown of dual oxidase 1 (DUOX1) promotes Wound Healing by regulating reactive oxygen species (ROS) by activation of Nuclear Kactor kappa B (NF-κB) signaling. Med Sci Monit.

[23] Wu L, Wang L, Du Y, Zhang Y, Ren J (2023). Mitochondrial quality control mechanisms as therapeutic targets in doxorubicin-induced cardiotoxicity. Trends Pharmacol Sci.

[24] Chen M, Qiao Y, Cao J, Ta L, Ci T, Ke X (2022). Biomimetic doxorubicin/ginsenoside co-loading nanosystem for chemoimmunotherapy of acute myeloid leukemia. J Nanobiotechnol.

[25] Song Y, Chen X, Huang R, Liu J (2023). Dysregulated YAP1/Hippo Pathway Contributes to Doxorubicin (ADM) Resistance in Acute myeloid leukemia (AML). Curr Pharm Biotechnol.

[26] Kciuk M, Gielecińska A, Mujwar S, Kołat D, Kałuzińska-Kołat Ż, Celik I, Kontek R (2023). Doxorubicin-an agent with multiple mechanisms of anticancer activity. Cells.

[27] Oh CM, Cho S, Jang JY, Kim H, Chun S, Choi M, Park S, Ko YG (2019). Cardioprotective potential of an SGLT2 inhibitor against doxorubicin-induced heart failure. Korean Circulation J.

[28] Zhang L, Feng Q, Wang T (2018). Necrostatin-1 protects against paraquat-induced cardiac contractile dysfunction via RIP1-RIP3-MLKL-Dependent necroptosis pathway. Cardiovasc Toxicol.

[29] Chopra K, Folkmanaitė M, Stockdale L, Shathish V, Ishibashi S, Bergin R, Amich J, Amaya E (2023). Duox is the primary NADPH oxidase responsible for ROS production during adult caudal fin regeneration in zebrafish. iScience.

[30] Ashtiwi NM, Sarr D, Rada B (2021). DUOX1 in mammalian disease pathophysiology. J Mol Med (Berl).

[31] Yang F, Qin Y, Wang Y, Li A, Lv J, Sun X, Che H, Han T, Meng S, Bai Y (2018). LncRNA KCNQ1OT1 mediates pyroptosis in diabetic cardiomyopathy. Cellular Physiology and Biochemistry.

[32] Han Y, Qiu H, Pei X, Fan Y, Tian H, Geng J (2018). Low-dose Sinapic Acid abates the pyroptosis of macrophages by downregulation of lncRNA-MALAT1 in rats with diabetic atherosclerosis. J Cardiovasc Pharmacol.

[33] Zeng C, Wang R, Tan H (2019). Role of pyroptosis in cardiovascular diseases and its therapeutic implications. Int J Biol Sci.

[34] Toldo S, Mezzaroma E, Buckley LF, Potere N, Di Nisio M, Biondi-Zoccai G, Van Tassell BW, Abbate A (2022). Targeting the NLRP3 inflammasome in cardiovascular diseases. Pharmacol Ther.

[35] Zheng Y, Xu L, Dong N, Li F (2022). NLRP3 inflammasome: the rising star in cardiovascular diseases. Front Cardiovasc Med.

[36] Shen S, Wang Z, Sun H, Ma L (2022). Role of NLRP3 inflammasome in myocardial ischemia-reperfusion injury and ventricular remodeling. Med Sci Monit.

[37] Wan T, Li X, Fu M, Gao X, Li P, Guo W (2022). NLRP3-Dependent pyroptosis: a candidate therapeutic target for Depression. Front Cell Neurosci.

[38] Abbate A, Toldo S, Marchetti C, Kron J, Van Tassell BW, Dinarello CA (2020). Interleukin-1 and the inflammasome as therapeutic targets in cardiovascular disease. Circ Res.

[39] Wu J, Dong E, Zhang Y, Xiao H (2021). The role of the inflammasome in heart failure. Front Physiol.

