# Supplementary material for: Upregulated dual oxidase 1-induced oxidative stress and caspase-1-dependent pyroptosis reflect the etiologies of heart failure
Source: BMC Mol Cell Biol. 2024 May 15;25:16. doi: 10.1186/s12860-024-00506-8 (PMC11094974; doi:10.1186/s12860-024-00506-8)

Figure 1

Duox1


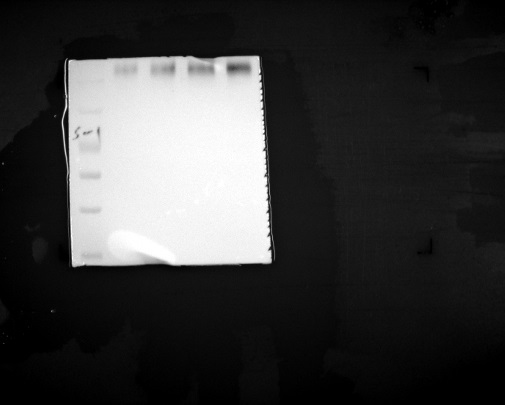


GAPDH


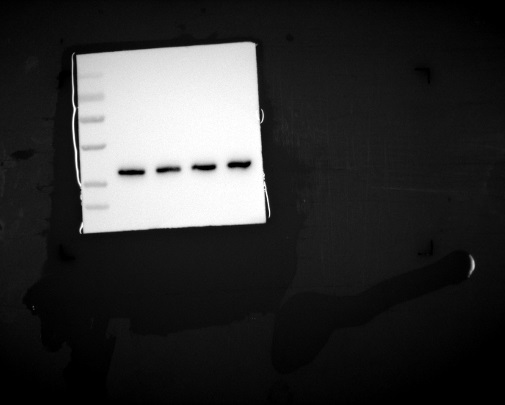


Figure 2 B

Duox1


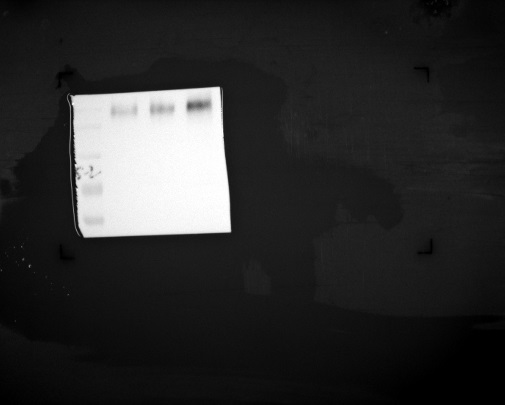


GAPDH


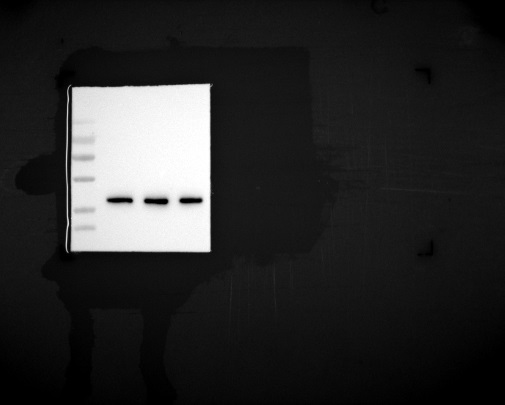


Figure 2 E

GAPDH


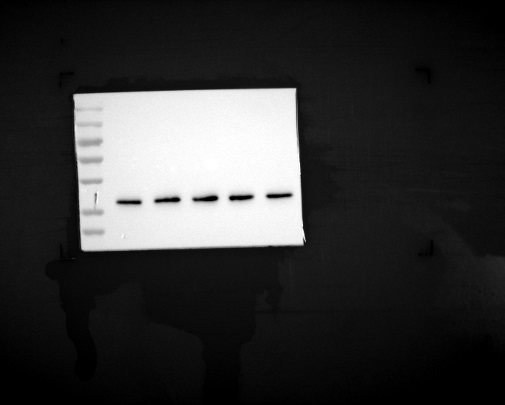


Duox1


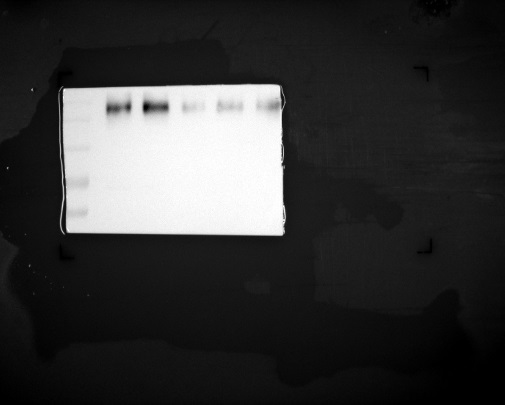


Figure 4

Pro-Casp-1


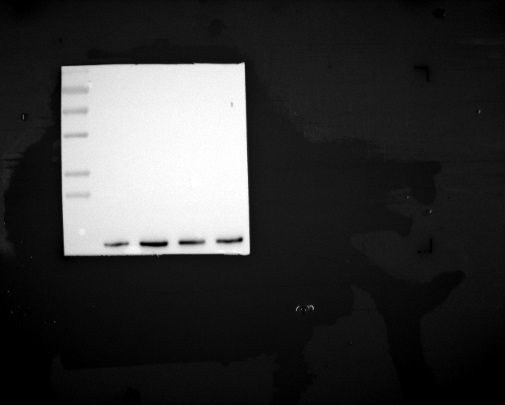


Active-Casp-1


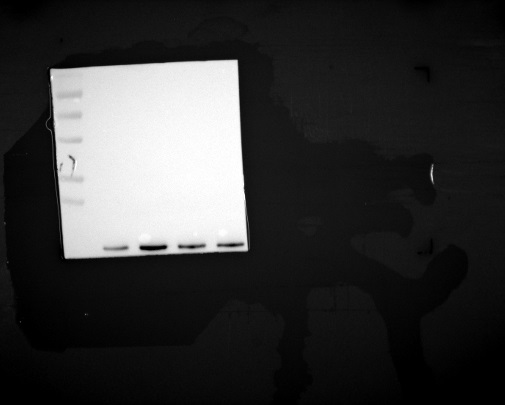


GAPDH


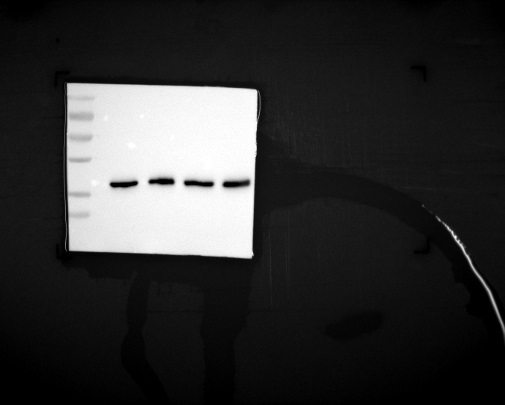


GSDMD-N


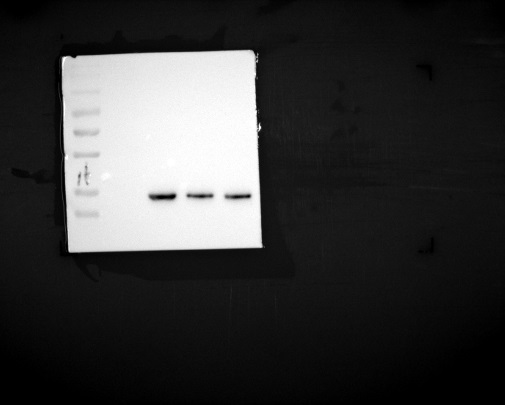


NLRP3


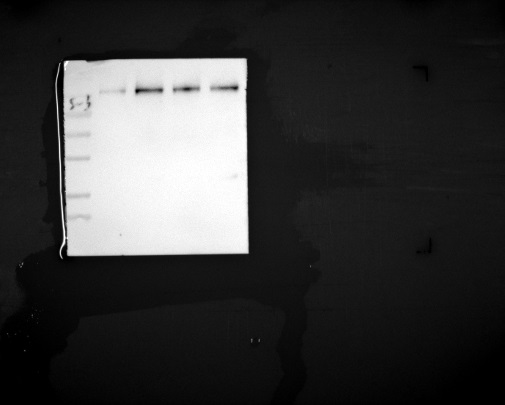


GAPDH


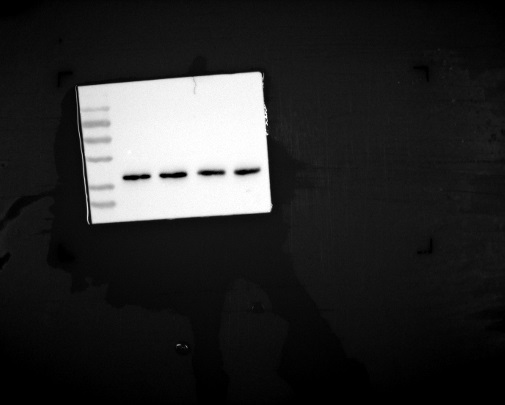


Figure 6

Pro-Casp-1


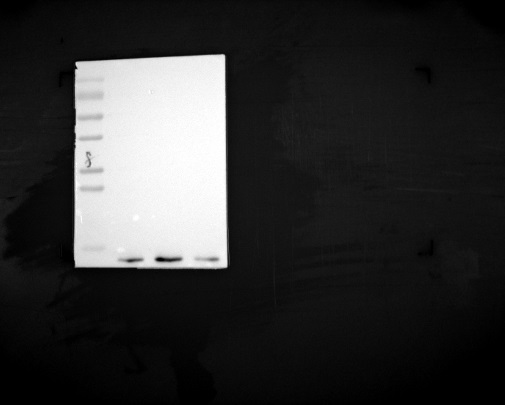


Active-Casp-1


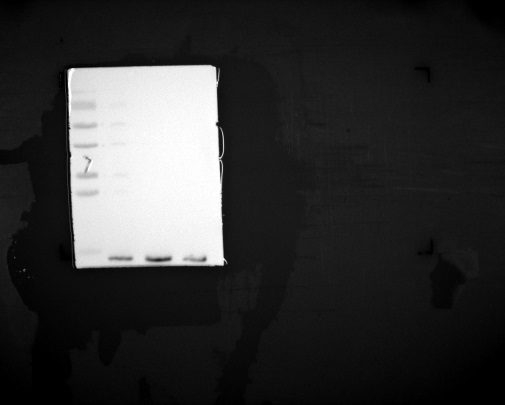


GAPDH


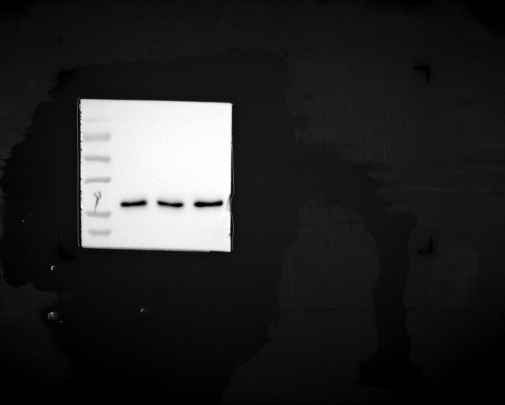


GSDMD-N


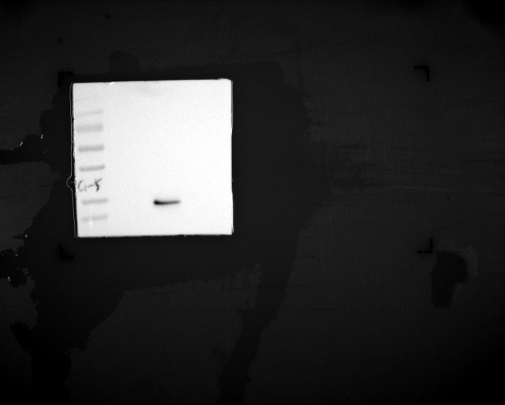


NLRP3


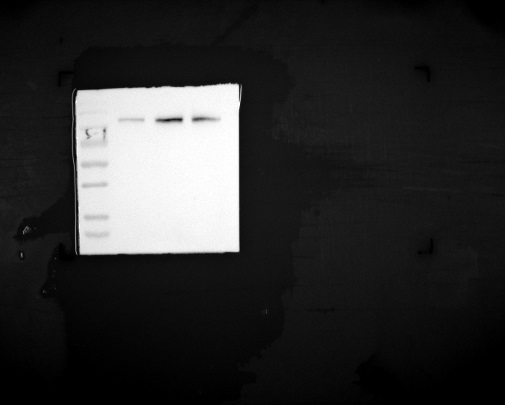


GAPDH


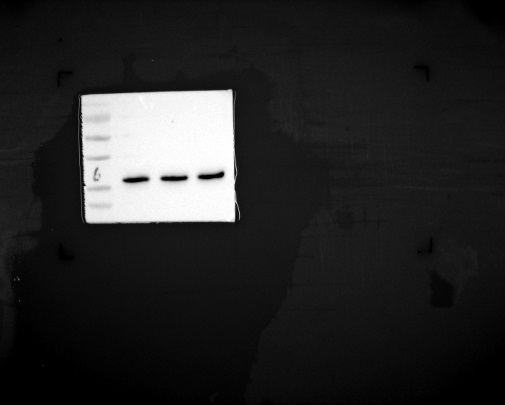

Supplement: Supplementary file 1 — Supplementary Material 1. [file 12860_2024_506_MOESM1_ESM.docx]
